# Remote analysis of auditory performance in cochlear implant users using a new tool: remote check

**DOI:** 10.1016/j.bjorl.2026.101902

**Published:** 2026-10-15

**Authors:** Francisco Wallison Lucena da Silva, Fernanda Ferreira Caldas, Carolina Costa Cardoso, Ingrid Barros da Silva Santana, Rayssa Pacheco Brito Dourado, Fayez Bahmad

**Affiliations:** a Universidade de Brasília (UnB), Brasília, DF, Brazil; b Instituto Brasiliense de Otorrinolaringologia, Brasília, DF, Brazil

**Keywords:** Auditory rehabilitation, Cochlear implant, Hearing Los, Remote check.

## Abstract

**Objective:**

To evaluate and characterize the auditory performance of Cochlear Implant (CI) users through Remote Check tool via mobile device.

**Methods:**

A prospective cross-sectional analytical study was carried out. Thirty-one cochlear implant users prelingual and postlingual participated in the study, with at least 6-months of implant use, without cognitive or intellectual changes associated with hearing loss. Remote Check was made available via the Nucleus Smart app and managed by the My Cochlear Professional platform. Participants responded to the test and data regarding Digit Triplets Test (DTT), Aided Threshold Test (ATT) and time of use were collected.

**Results:**

Comparative tests indicated significant differences in speech-in-noise levels between groups of people with post-lingual and pre-lingual hearing loss. As for the hearing thresholds, significant difference was observed at different frequencies, varying according to use. In addition, the analysis of effect size showed moderate differences in speech in noise and some frequencies, and high effect at 250 Hz on left ear and device usage time for those wearing on right ear.

**Conclusion:**

Remote Check is an effective tool that can assist in describing the profile of cochlear implant users. It was possible to trace the profile of CI users in the stage of datalogging, assessment of auditory thresholds and in speech-in-noise tests.

**Level of evidence:**

2 (Step 2).

## Highlights

• Remote Check is feasible for describing the auditory profile of Cochlear Implant users.
• Remote Check can assist in the remote monitoring of Cochlear Implant users.
• Remote Check is a tool that can be used in clinical practice by the audiologist.

## Introduction

Cochlear Implant (CI) is an electronic device surgically inserted to restore hearing in individuals with severe to profound hearing loss who no longer benefit from hearing aids.^1^ CI use improves speech recognition, social interaction, cognitive stability, and overall quality of life.^2^ Individual auditory outcomes vary according to age, duration of deafness, residual hearing, and prior use of hearing aids.^3^ Cognitive aspects, particularly short-term memory, are also associated with post-implant performance.^4^

During the initial months of CI programming, the goal is to ensure audibility and user adaptation, with stable adjustments typically achieved after one year.^5^ Clinical assessment traditionally relies on tests conducted in an acoustic booth, such as Pure-Tone Audiometry (PTA) and Speech Recognition Threshold (SRT).^6^ However, hearing evaluation can also be conducted through Direct Audio Input (DAI) or Digital Audio Streaming (DAS), allowing signal transmission without an acoustic booth.^7^

Remote monitoring has become a valuable alternative, enabling distance follow-up of CI users. Remote Check (RC), developed by Cochlear™ and available within the Nucleus Smart App, provides a set of self-administered tests, questionnaires, and photo documentation, allowing patients or caregivers to perform assessments at home.^8^ These data are supervised by speech-language pathologists through the My Cochlear Professional (MCP) platform.

This study aimed to evaluate and characterize the auditory performance of unilateral and bilateral CI users through Remote Check using mobile devices.

## Methods

This prospective cross-sectional analytical study was approved by the Ethics Committee (CAAE: 83476324.5.0000.5558). All participants provided informed consent. The research was conducted at a CI reference center in Faculty of Medicine of University of Brasília between September and December 2024.

Thirty-one CI users participated. Inclusion criteria comprised absence of cognitive deficits, at least six months of CI experience, and use of Nucleus 7, Nucleus 8, or Kanso 2 processors with Bluetooth connectivity. Remote Check was managed via My Cochlear Professional, an online platform that allows professionals to send and monitor remote assessments. Participants received instructions via WhatsApp on how to perform the tests (estimated duration 20–40 min) and were encouraged to complete them in a quiet environment. Assistance was available through messaging or in person. Upon completion, data collection was conducted by a single audiologist and individual reports were automatically generated through MCP and organized in spreadsheets for analysis.

### Remote check components

Remote Check includes Implant Site Photos, Questionnaire, Aided Threshold Test (ATT), Digit Triplet Test (DTT), Impedance Check, and Usage Data. For this study, four components were analyzed:

• Usage Data: Logs on device-on time, volume, sensitivity, program use, and exposure to auditory environments.
• Digit Triplet Test (DTT): Determines speech perception in noise by presenting random digit sequences (1–9) at varying Signal-to-Noise Ratios (SNRs). Participants select the heard digits on the screen.
• Aided Threshold Test (ATT): Direct pure-tone stimuli (250 Hz–6 kHz) are streamed to the CI, and users indicate whether tones are audible or not, generating an audiogram.

### Statistical analysis

Analyses were conducted using JASP. Descriptive statistics summarized categorical and numerical variables. Normality was assessed using the Shapiro–Wilk test.^9^ Independent-sample *t*-tests were applied for normally distributed data, and Mann–Whitney tests for non-normal data.^10^ Effect sizes were calculated using Cohen’s *d* or rank-biserial correlation, interpreted as small (≤0.2), moderate (0.2–0.5), or large (>0.5).^11^

## Results

Remote Check procedures of each patient were completed during the estimated 20−40 min, with no additional time.

A total of 31 participants were included in the study. 11 participants were bilateral Cochlear Implant (CI) users (35.48%), whereas 20 were unilateral CI users (64.52%). Among the unilateral CI users, 18 had hearing loss in the contralateral ear. Of these, 8 had bimodal hearing (CI in one ear and a hearing aid in the other). 5 participants had undergone bilateral cochlear implantation but were using only one implant at the time of assessment. The remaining 5 participants did not use any hearing device in the contralateral ear despite meeting clinical indications for cochlear implantation. Only 2 participants presented with Single-Sided Deafness (SSD).

Participants' duration of CI use ranged from 2-years and 2-months to 24-years.

The mean age was 48.16-years, ranging from 15 to 79. Among the unilateral users, 11 had the implant on the left side (55.00%) and 9 on the right side (45.00%). Considering all participants, most of the sample was female (61.29%). In the bilateral CI user group, females accounted for 63.64%, while in the unilateral group, they represented 60.00%. Focusing only on the unilateral CI group, females made up 77.78% of those with a right-ear implant, while males represented 55.55% of those with a left-ear implant. Regarding the type of hearing loss, most cases were postlingual (64.52%), while prelingual cases accounted for 35.48%. The results are shown in Table 1.

**Table 1:** Descriptive statistics of categorical variables.

		Bilateral CI	Unilateral CI (Right)	Unilateral CI (Left)	Total
Sex	Female	7 (63.64%)	7 (77.78%)	5 (45.45%)	19 (61.29%)
	Male	4 (36.36%)	2 (22.22%)	6 (55.55%)	12 (38.71%)
Onset of Hearing Loss	Prelingual	6 (54.55%)	1 (11.11%)	4 (36.36%)	11 (35.48%)
	Poslingual	5 (45.45%)	8 (88.89%)	7 (63.64%)	20 (64.52%)

When comparing prelingual and postlingual hearing loss groups, the descriptive results are presented in Table 2.

**Table 2:** Descriptive results in dB HL for prelingual and postlingual groups in speech-in-noise performance.

	Group	M	SD	Q1	Q2	Q3	Min	Max	p
Bilateral (RE)	Prelingual	−0.90	‒	−0.90	−0.90	−0.90	−0.90	−0.90	‒
	Prelingual	−0.75	2.88	−2.78	−0.80	1.03	−4.30	3.20	0.64
Bilateral (LE)	Prelingual	−2.77	2.14	−3.55	−1.90	−1.55	−5.20	−1.20	0.31
	Prelingual	0.94	1.80	−0.70	1.50	1.80	−1.10	3.20	0.54
Unilateral (RE)	Prelingual	−0.63	1.47	−1.25	−0.35	0.28	−2.60	0.80	0.74
	Prelingual	−3.62	1.43	−4.60	−4.20	−2.20	−5.10	−2.00	0.22
Unilateral (LE)	Prelingual	0.05	1.42	−0.78	−0.25	0.58	−1.30	2.00	0.65

dB HL, Decibels Hearing Level; RE, Right Ear; LE, Left Ear; SD, Standard Deviation; Q1-Q3, Quartiles; M, Mean; Min, Minimum value; Max, Maximum value; p, Significance level from the Shapiro–Wilk test; p < 0.05.

Although some groups showed non-significant results in the Shapiro–Wilk test (indicating a normal distribution), nonparametric tests (Mann–Whitney) were chosen for the comparative analysis due to the number of participants in each group. The comparative tests indicated significant differences in speech-in-noise levels for left-ear between the groups with postlingual and prelingual hearing loss. This difference applies to both bilaterally and unilaterally implanted individuals (Table 3).

**Table 3:** Results of inferential analyses between unilateral and bilateral groups for speech-in-noise performance.

	Side	U	p	rrb
Bilateral	Right Ear	18	0.063	−0.800
	Left Ear	19	0.037	−0.900
Unilateral	Right Ear	‒	‒	‒
	Left Ear	0	0.036*	1.000

dB HL, *U*: Mann–Whitney test statistic; p, Significance level; *r*<sub > rb</sub>, Rank-biserial correlation (effect size estimate for the Mann–Whitney test); p < 0.05.

Among users with unilateral cochlear implant, individuals with prelingual hearing loss showed poorer results compared to those with postlingual hearing loss. On the other hand, individuals with prelingual hearing loss in the group of bilateral cochlear implant users showed better results compared to participants with postlingual hearing loss in the same group.

Table 4, Table 5 presents descriptive statistics for numerical variables corresponding to the results at each sound frequency tested via the Remote Check. Auditory threshold assessments using Pure-Tone Audiometry (PTA), initially considering only bilateral cochlear implant users, revealed significant difference at 1 kHz, 4 kHz, and 6 kHz in the right ear, and at 1 kHz and 6 kHz in the left ear. In the unilateral cochlear implant group, significant difference were observed at 1 kHz, 2 kHz, 3 kHz, and 6 kHz among users with implants exclusively in the right ear. In contrast, users with unilateral implants in the left ear exhibited significant difference at 500 Hz, 1 kHz, 4 kHz, and 6 kHz.

**Table 4:** Right ear auditory thresholds (dB HL) by frequency in the unilateral and bilateral CI groups.

		M	SD	Q1	Q2	Q3	Min	Max	p
250 Hz	Bi	19.03	6.04	14.91	17.66	21.08	12.83	33.50	0.058
	Uni	21.66	5.12	20.58	20.83	21.08	15.50	31.17	0.061
500 Hz	Bi	18.63	5.69	16.25	18.33	18.74	11.17	30.50	0.206
	Uni	18.98	5.35	16.67	18.17	20.16	11.38	28.83	0.552
1 KHz	Bi	17.10	5.28	14.25	15.50	17.49	11.50	27.17	**0.029**
	Uni	16.16	4.28	14.41	14.83	15.83	12.86	26.50	**<0.001**
2 KHz	Bi	18.42	5.57	14.17	18.00	21.66	10.33	26.21	0.184
	Uni	19.06	6.16	14.33	17.50	21.50	13.50	33.00	**0.045**
3 KHz	Bi	18.75	4.28	15.50	17.50	19.50	14.50	26.83	0.125
	Uni	17.51	3.88	15.25	17.01	18.20	13.50	27.50	**0.008**
4 KHz	Bi	17.64	6.70	15.17	16.17	16.83	12.10	34.50	**0.001**
	Uni	15.50	4.29	13.83	15.50	16.50	10.50	25.50	0.052
6 KHz	Bi	17.27	6.25	14.50	15.50	17.17	12.50	32.83	**0.002**
	Uni	17.33	6.23	14.41	15.50	16.49	12.50	32.17	**0.001**

dB HL, Decibels Hearing Level; Bi, Bilateral; Uni, Unilateral; SD, Standard Deviation; Q1‒Q3, Quartiles; M, Mean; Min, Minimum value; Max, Maximum value; p, Significance level from the Shapiro–Wilk test; p < 0.05.

**Table 5:** Left ear auditory thresholds (dB HL) by frequency in the unilateral and bilateral CI groups.

		M	SD	Q1	Q2	Q3	Min	Max	p
250 Hz	Bi	18.86	2.74	16.65	19.83	21.33	14.50	21.50	0.065
	Uni	20.53	1.85	20.50	21.17	21.50	17.50	22.83	0.067
500 Hz	Bi	17.73	2.30	16.16	18.50	18.50	13.50	21.50	0.709
	Uni	17.46	2.39	16.25	18.50	18.74	12.17	19.50	**0.008**
1 KHz	Bi	15.98	2.51	14.50	15.50	16.17	13.50	22.17	**0.008**
	Uni	16.50	2.82	15.41	15.66	16.50	13.83	23.17	**0.002**
2 KHz	Bi	14.03	1.50	14.17	14.50	14.50	11.50	15.50	0.141
	Uni	14.78	2.69	13.33	14.50	16.00	11.17	19.17	0.864
3 KHz	Bi	15.63	1.19	15.50	15.50	15.50	14.17	17.50	0.238
	Uni	15.78	3.18	14.00	15.50	16.66	12.17	21.50	0.365
4 KHz	Bi	15.43	1.57	15.50	15.50	16.50	12.83	16.83	0.200
	Uni	16.39	7.33	11.50	15.50	16.50	11.17	32.09	**0.006**
6 KHz	Bi	14.43	1.47	13.17	15.50	15.50	12.50	15.50	**0.028**
	Uni	19.42	13.51	13.83	14.83	15.33	12.83	50.00	**<0.001**

dB HL, Decibels Hearing Level; Bi, Bilateral; Uni, Unilateral; SD, Standard Deviation; Q1-Q3, Quartiles; M, Mean; Min, Minimum value; Max, Maximum value; p, Significance level from the Shapiro–Wilk test; p < 0.05.

Table 6 presents the descriptive statistics of numerical variables related to device usage time by ear for both unilateral and bilateral cochlear implant users.

**Table 6:** Datalogging of CI users in unilateral and bilateral groups.

	Side	M	SD	Q1	Q2	Q3	Min	Max	p
Bilateral	Right Ear	9.35	5.06	6.27	10.55	13.47	1.20	14.70	0.153
	Left Ear	10.31	4.46	8.17	11.40	13.90	1.20	15.00	0.246
Unilateral	Right Ear	11.71	3.71	10.20	11.35	14.22	6.10	16.50	0.908
	Left Ear	9.66	4.83	6.00	10.90	12.30	0.30	15.00	0.355

SD, Standard Deviation; Q1-Q3, Quartiles; M, Mean; Min, Minimum value; Max, Maximum value; p, Significance level from the Shapiro–Wilk test; p < 0.05.

Table 7 presents the descriptive statistics of the numerical variables related to performance in decibels (dB) on the Digit Triplet Test for each ear, for both unilateral and bilateral cochlear implant users.

**Table 7:** Auditory performance in dB HL on the digit triplet test for unilateral and bilateral CI users.

	Side	M	DP	Q1	Q2	Q3	Min	Max	p
Bilateral	Right Ear	2.28	1.30	1.30	2.45	3.17	0.40	4.40	0.794
	Left Ear	2.63	1.22	1.87	2.85	3.30	0.50	4.60	0.912
Unilateral	Right Ear	2.72	0.88	2.20	2.60	2.90	1.80	4.50	0.177
	Left Ear	2.86	1.61	1.50	2.75	4.22	0.90	5.00	0.347

SD, Standard Deviation; Q1-Q3, Quartiles; M, Mean; Min, Minimum value; Max, Maximum value; p, Significance level from the Shapiro–Wilk test; p < 0.05.

Regarding the inferential comparative tests, no differences were observed between groups when comparing unilateral and bilateral cochlear implant users, using Student’s *t*-tests for normally distributed data and Mann–Whitney tests for non-normally distributed data. The results can be found in detail in Table 8, Table 9. Effect size analysis indicated moderate differences between groups for the speech-in-noise variables in the right ear. Moderate effect sizes were also observed at frequencies of 250 Hz, 3 kHz, and 4 kHz in the right ear, and at 2 kHz in the left ear. Additionally, the variable at 250 Hz in the left ear and the usage time of the device in the right ear demonstrated differences with large effect sizes.

**Table 8:** Right Ear results of inferential analyses between unilateral and bilateral groups.

	Statistic	df	p	Effect Size (r)	Interpretation
Usage Time^a^	−0.991	14	0.339	−0.512	Large
Speech in Noise^a^	−0.788	15	0.443	−0.388	Moderate
250 Hz^a^	−0.889	14	0.389	−0.459	Moderate
500 Hz^a^	−0.134	16	0.895	−0.063	Low
1 KHz^b^	43.000	‒	0.824	0.075	Low
2 KHz^a^	−0.248	19	0.807	−0.108	Low
3 KHz^a^	0.662	17	0.517	0.304	Moderate
4 KHz^a^	0.807	16	0.432	0.380	Moderate
6 KHz^b^	35.500	‒	1.000	−0.014	Low

^a^ Student’s *t*-test; ^b^ Mann–Whitney test; Statistic, Test statistic used; df, Degrees of freedom; p, Significance level of the comparative test; Effect Size, Effect size estimate, Cohen’s *d* for the Student’s *t*-test and point-biserial correlation for the Mann–Whitney test.

**Table 9:** Left Ear results of inferential analyses between unilateral and bilateral groups.

	Statistic	df	p	Effect Size (r)	Interpretation
Usage Time^a^	0.302	17	0.767	0.139	Low
Speech in Noise^a^	−0.349	16	0.732	−0.165	Low
250 Hz^a^	−1.542	17	0.142	−0.708	Large
500 Hz^a^	0.253	18	0.803	0.113	Low
1 KHz^b^	30.500	‒	0.627	−0.153	Low
2 KHz^a^	−0.560	10	0.588	−0.328	Moderate
3 KHz^a^	−0.100	10	0.922	−0.059	Low
4 KHz^a^	−0.285	10	0.781	−0.167	Low
6 KHz^b^	18.000	‒	1.000	0.029	Low

^a^ Student’s *t*-test; ^b^ Mann–Whitney test; Statistic, Test statistic used; df, Degrees of freedom; p, Significance level of the comparative test; Effect Size, Effect size estimate, Cohen’s *d* for the Student’s *t*-test and point-biserial correlation for the Mann–Whitney test.

## Discussion

The application of Remote Check proved to be positive by presenting a novel approach for patient monitoring in clinical practice.

Based on our study, 11 individuals with prelingual hearing loss and 20 with postlingual hearing loss were identified. When comparing individuals using bilateral CIs, prelingual participants demonstrated better performance on the speech-in-noise test. According to Bugannim,^12^ speech understanding in noise can be achieved by cochlear implant users with pre-lingual hearing loss, provided that this ability is developed through auditory training, potentially reaching performance levels comparable or close to those of normal-hearing individuals.

Regarding the prelingual group, it is known that the prognosis after implantation can vary across multiple factors. In the study by Forli,^13^ for example, participants were tested post-implantation using disyllabic word recognition tasks and obtained results ranging from 0% to 100% in quiet and from 0% to 80% in noise. Such variability in performance can be attributed to a range of factors, including the age at onset of hearing loss, age at initiation of hearing aid use, residual hearing, education level, motivation, and psychological aspects.

For postlingual hearing loss, the present study demonstrated better performance in speech-in-noise tests for individuals using unilateral cochlear implant. In these cases, according to Jaiswal,^14^ in a study conducted with 21 individuals with severe to profound bilateral post-lingual hearing loss, the findings showed significant improvement in speech intelligibility for patients who were implanted even many years after the onset of deafness, reinforcing that implantation in postlingual adults can yield favorable outcomes. Another factor contributing to good auditory performance in noisy environments may also be related to auditory memory, which, according to Prince,^15^ besides enhancing speech intelligibility, may extend its benefits to broader aspects of quality of life. When investigating quality of life, Rostkowska^16^ found results indicating that cochlear implants can also benefit this population by improving aspects such as independence, mental health, happiness, self-esteem, relationships, and others.

For the assessment of auditory thresholds between individuals with unilateral and bilateral cochlear implants, significant difference was observed at certain frequencies for both groups, as well as moderate to high effects at specific frequencies. In other words, a noticeable and relevant difference was found between the tested groups, which, in practical terms, may become more perceptible with an increased sample size and greater homogeneity between groups.

According to Sorrentino,^17^ Remote Check is an important tool that can be used to assess auditory thresholds and may also be useful for comparing frequency-specific auditory thresholds in acoustic booth settings, allowing for testing of the cochlear implant microphone’s functionality, which cannot be evaluated via Remote Check. Additionally, the microphone may deteriorate over time due to dust, damage, or moisture, potentially leading to reduced performance.

This study also found that the mean auditory thresholds obtained via Remote Check (RC) ranged from 14.03 Db to 21.66 dB. This result demonstrates that cochlear implant users exhibit lower auditory thresholds, which is favorable, as a lower intensity level in dB facilitates access to softer sounds due to the electrical stimulation provided by the CI combined with the mapping adjustment parameters.^18^^,^^19^ This finding is also supported by Chang,^20^ whose study with 20 implanted adults showed improved speech perception when auditory thresholds were between 25 and 35 dB.

Lower auditory thresholds not only facilitate access to softer sounds but also allow cochlear implant users to reduce listening fatigue and effort, provided these aspects are addressed in auditory training.^21^ Along with the reduction in listening effort, the resulting benefits can be observed as improved listening performance both in quiet conditions and in noisy environments.^22^

These data can guide the audiologist in establishing more precise protocols when mapping the cochlear implant of their patients, aiming to optimize the parameters to enhance its use and performance. In addition, teleconsultation can be a viable option to assess the need for adjustment changes in individual cases before scheduling an in-person appointment to carry out the procedure.^23^

Regarding Cochlear Implant (CI) usage time, when analyzing usage per ear among participants with bilateral CIs, the average usage time was 9.35-hs for the right ear and 10.31-hs for the left ear. Among participants using a CI in only one ear, the average usage time was 11.71-hs for the right ear and 9.66-hs for the left ear. Busch^24^ analyzed datalogging information from 1,501 patients and found an average CI usage time of 10.7-hs, which is consistent with our findings and supports the recommendation that daily CI processor use of at least 10-hs per day leads to better outcomes.

In the study by Lindquist,^25^ an average CI usage time of 12-hs per day was found for most adults during the first year after activation, which resulted in better speech comprehension performance compared to those who did not use the device consistently. Such findings may be relevant for decision-making and for providing guidance on strategies to further increase cochlear implant usage time.

In terms of the Digit Triplets Test (DTT), which aims to assess speech comprehension in noise, the average Signal-to-Noise Ratio (SNR) performance ranged from 2.28 dB to 2.86 dB, with a minimum of 0.4 dB and a maximum of 5 dB. In a study conducted by Maruthurkkara^8^ with 32 participants, the mean DTT result was 1.6 dB, which supports our findings regarding the ease with which participants were able to handle the test and achieve the reported performance levels. According to Rosendahl,^26^ one factor that may explain the participants’ facility in performing this task is that the DTT is a closed-set test, meaning that the response options are limited: the digits that could appear in the three-digit sequences ranged only from 0 to 9. Another justification, according to Kaandorp,^27^ is that beyond being a closed-set task, the test consists of familiar and easily recognizable words, allowing the DTT to be used as a speech recognition test for CI users who are unable to perform more complex tasks.

The scoping review by Van den Borre^28^ on recent studies in this field supports the notion that the Digit Triplets Test (DTT) is a reliable and valid tool for remotely measuring auditory performance and listening abilities in cochlear implant users, in addition to allowing its application in a diverse population with varying characteristics.

Despite these results, there is a scarcity of studies in the literature that investigate the association of the Digit Triplets Test (DTT) with cochlear implants in a remote context. Cullington,^29^ when testing whether the DTT could be an effective and acceptable method for assessing speech recognition in CI users under conditions similar to a home environment, concluded that most CI users who participated in the study were able to complete the DTT to assess their speech recognition in a simulated home setting. Moreover, the test outcomes showed a strong correlation with other assessments involving sentence comprehension both in quiet and in adaptive noise conditions in clinical settings. In addition, compared with in-person testing, the participants in that study found the remote procedure more practical due to time optimization and the elimination of the need to travel to the clinic.

The literature presents several methods for assessing speech comprehension in Cochlear Implant (CI) users in the presence of competing noise. One such method is the Hearing In Noise Test (HINT), a validated test available in multiple languages with standardized procedures, which evaluates speech recognition both in quiet and in the presence of competing noise using recorded male-voice sentences presented to the patient in a sound booth.^30^ Another test used to assess speech comprehension in noise is the AzBio Sentence Test, which employs longer sentences with fewer contextual cues and recordings from multiple talkers to increase the level of difficulty.^31^ Willberg,^32^ in turn, introduced additional tests that may contribute to the assessment of this ability, namely the Finnish Matrix Sentence Test (FMST), Finnish Simplified Matrix Sentence Test (FINSIMAT), and Finnish Digit Triplet Test (FDTT). The latter can be administered remotely, as responses can be easily recorded using an image of a numeric keypad on a touchscreen device, similar to the Remote Check procedure. Therefore, as a way to complement the remote administration of the DTT via Remote Check, other tests simulating listening and speech comprehension in noise beyond digit-based stimuli could be incorporated into the platform to provide more comprehensive results and allow comparisons across additional domains.

When comparing usage performance between unilateral and bilateral CI users, no statistically significant difference was found, despite the effect size varying from moderate to large across the compared conditions. One possible explanation for this result, as suggested by Philips,^21^ may be the sample variability, which differed in number and age range between the participant groups. Therefore, further studies are recommended to reaffirm the importance of Remote Check as a tool for remote monitoring of cochlear implant users.

Regarding the general use of the RC, some patients may benefit more from its utilization. Individuals who have been using CI for over one year tend to present stable auditory outcomes and typically attend annual appointments primarily for implant monitoring. For this population, the use of Remote Check can serve as a solution for certain routine consultations, allowing patients to extend the interval between in-person visits. In cases of remote follow-up where a critical issue is detected via RC, the clinician can provide guidance or request an in-person clinical evaluation, as is also recommended for patients with special needs.^33^

On the other hand, despite the benefits of Remote Check, some limitations should also be considered. According to Sucher,^34^ the educational level of CI users may interfere with the completion of remote tests due to a lack of familiarity with computers and smartphones, as well as concerns and doubts related to the privacy and security of the tool. Consequently, audiologists may be reluctant to recommend this function to this population.

Another consideration is the possible need for a dedicated professional responsible for ensuring the effective execution of the procedure. This would guarantee that CI users complete all steps of the Remote Check tool, as well as verify that the data have been properly collected in the performance report.^35^

Nonetheless, Remote Check can be regarded as a valuable tool to be incorporated into clinical practice by audiologists working in auditory rehabilitation and cochlear implantation. The use of Remote Check has the potential to optimize both the clinician’s workflow and facilitate device usage for CI users in their daily routines. The instrument can complement the audiologist’s work by optimizing appointments and enabling remote monitoring of these individuals in cases where in-person consultations are not feasible for various reasons.

Future evidence-based investigations should be conducted to further support the use of Remote Check in clinical routines.

## Final considerations

The study had some limitations: participants were not stratified by age group, degree of hearing loss, and educational level was not considered to assess its impact on Remote Check performance. The sample size was limited, as many invited individuals did not complete the process due to mobile connectivity issues. No validated questionnaire was applied to participants after Remote Check to obtain their level of acceptance of the tool. Additionally, the tool does not assess binaural performance, since it evaluates only one ear at a time, even for bilateral cochlear implant users.

## Conclusion

Remote Check is an effective tool that can assist in profiling Cochlear Implant (CI) users. Through this tool, it is possible to characterize CI users both in the assessment of auditory thresholds and in speech understanding in noise. The tool can also help to monitor and document patient's progress throughout their daily routine.

## ORCID ID

Fernanda Ferreira Caldas: 0000-0003-3728-779X

## Financial support

This research did not receive any specific grant from funding agencies in the public, commercial, or not-for-profit sectors.

## Data availability statement

The authors declare that all data are available in repository.

## Declaration of competing interest

The authors declare no conflicts of interest.
